# Altered molecular pathways and prognostic markers in active systemic juvenile idiopathic arthritis: Integrated bioinformatic analysis

**DOI:** 10.17305/bjbms.2021.6016

**Published:** 2021-09-03

**Authors:** Yi Ren, Hannah Labinsky, Andriko Palmowski, Henrik Bäcker, Michael Müller, Arne Kienzle

**Affiliations:** 1 Center for Musculoskeletal Surgery, Charité – Universitätsmedizin Berlin, Corporate Member of Freie Universität Berlin, Humboldt-Universität Zu Berlin, and Berlin Institute of Health, Berlin, Germany; 2 Department of Internal Medicine, Rheumatology and Immunology, Friedrich-Alexander-University (FAU) Erlangen-Nürnberg and University Hospital Erlangen, Erlangen, Germany; 3 Department of Rheumatology and Clinical Immunology, Charité – Universitätsmedizin Berlin, Corporate Member of Freie Universität Berlin, Humboldt-Universität zu Berlin, and Berlin Institute of Health, Berlin, Germany; 4 Laboratory of Adaptive and Regenerative Biology, Brigham and Women’s Hospital, Harvard Medical School, Boston MA, USA

**Keywords:** Systemic juvenile idiopathic arthritis, hub genes, neutrophil, prognostic marker

## Abstract

Systemic juvenile idiopathic arthritis (SJIA) is a severe childhood-onset inflammatory disease characterized by arthritis accompanied by systemic auto-inflammation and extra-articular symptoms. While recent advances have unraveled a range of risk factors, the pathomechanisms involved in SJIA and potential prognostic markers for treatment success remain partly unknown. In this study, we included 70 active SJIA and 55 healthy control patients from the National Center for Biotechnology Information to analyze for differentially expressed genes (DEGs) using R. Functional enrichment analysis, protein-protein interaction (PPI), and gene module construction were performed for DEGs and hub gene set. We additionally examined immune system cell composition with CIBERSORT and predicted prognostic markers and potential treatment drugs for SJIA. In total, 94 upregulated and 24 downregulated DEGs were identified. Two specific modules of interest and eight hub genes (*ARG1*, *DEFA4*, *HP*, *MMP8*, *MMP9*, *MPO*, *OLFM4*, and *PGLYRP1*) were screened out. Functional enrichment analysis suggested that complex neutrophil-related functions play a decisive role in the disease pathogenesis. CIBERSORT indicated neutrophils, M0 macrophages, CD8+ T cells, and naïve B cells to be relevant drivers of disease progression. In addition, we identified *TPM2* and *GZMB* as potential prognostic markers for treatment response to canakinumab. Moreover, sulindac sulfide, (-)-catechin, and phenanthridinone were identified as promising treatment agents. This study provides a new insight into molecular and cellular pathogenesis of active SJIA and highlights potential targets for further research.

## INTRODUCTION

Systemic juvenile idiopathic arthritis (SJIA) is a childhood-onset inflammatory disease that is characterized by arthritis accompanied by systemic auto-inflammation and extra-articular symptoms. Disease burden is often high and therapeutic options limited, making diseases treatment highly challenging for patients and physicians alike. While recent advances have unraveled a range of risk factors, the pathomechanisms involved in SJIA remain complex and partly unknown [1,2].

Besides arthritis, SJIA is characterized by quotidian fever of ≥39°C that persists for longer than 2 weeks, evanescent erythematous skin rashes, and at least one of the following clinical features: Lymphadenopathy, pericarditis, pleuritis, or hepatosplenomegaly [3]. In addition, approximately 10% of all patients are prone to developing macrophage activation syndrome (MAS), a life-threatening complication [4,5].

Disease activity in affected patients can vary greatly and may range from clinically inactive to high disease activity [6,7]. Recent advances have identified prominent pro-inflammatory activation independent of disease activity [6]. In particular, patients present with leukocytosis, thrombocytosis, and highly elevated erythrocyte sedimentation rate and C-reactive protein concentration. In contrast to other subtypes of juvenile idiopathic arthritis, dysregulation of the innate immune system plays a significant role in disease progression [8]. In SJIA, toll-like receptor (TLR) signaling pathways mediate aberrant activation of phagocytes including monocytes, macrophages, and neutrophils [9]. These key players in the innate immune system are responsible for the subsequent release of pivotal pro-inflammatory cytokines [9]. In particular, expression of interleukin-1b, interleukin-6, and interleukin-18 was found to be significantly elevated in SJIA, leading to current targeted treatment approaches with biologicals such as anakinra or tocilizumab [10,11]. However, high rates of refractory and recurrent disease suggest other pathways to be involved in SIJA [4].

The use of traditional methods such as polymerase chain reaction, immunohistochemistry, and flow cytometry for immune system composition is not optimal for high throughput. Microarray technology, a powerful strategy to test expression of thousands of genes simultaneously, has widely gained attention for the profiling of differentially expressed genes (DEGs). Bioinformatic analysis on transcription level is capable of defining hub genes, significant signaling pathways, and immune composition patterns. Comprehensive evaluation of the involved immune cells may offer new treatment approaches for affected patients.

In this study, we analyzed immune cell composition, key genes, pathways, and protein-protein interactions in active SJIA by utilizing a bioinformatic analysis method. Subsequently, we analyzed potential drugs as new treatment approaches based on identified DEGs.

## MATERIALS AND METHODS

### Microarray data

All primary data analyzed in this study were accessed from public data repositories. Dataset GSE17590 and GSE80060 were downloaded from Gene Expression Omnibus (GEO) database (http://www.ncbi.nlm.nih.gov/geo/) of the National Center for Biotechnology Information. The datasets consist of gene expression data of whole peripheral blood samples from 44 patients diagnosed with active SJIA (placebo treated), from 43 healthy control patients, and from 77 patients treated with canakinumab [12,13]. Active SJIA was defined by a juvenile arthritis disease activity score (JADAS) above 8.5, laboratory parameters (C-reactive protein and erythrocyte sediment rate), and overt disease symptoms. Besides, we also obtained GSE112057, a RNA-sequencing high-throughput dataset, as a verification cohort with a total of 38 patients (26 active SJIA patients and 12 healthy controls) [14].

### Primary data processing and identification of DEGs

Raw data from GSE17590 and GSE80060 were loaded into R software (R Development Core Team; version: 3.6.3). After combining the expression matrices of these two datasets, the interbatch difference was removed using the “sva” package [15]. Results before and after batch effect removal were plotted using a two-dimensional PCA cluster graph. “limma” package was employed to identify differences in DEGs in SJIA and in healthy controls [16]. Cutoff values for DEGs were set as adjusted *p* < 0.05 and |log2 fold change (FC)| >1.5. DEGs were visualized as heatmap and volcano plots using R packages “pheatmap” and “ggplot2” [17,18].

### Functional and pathway enrichment analysis

Gene ontology (GO) and Kyoto Encyclopedia of Genes and Genomes (KEGG) pathway enrichment analysis for DEGs were performed using the “clusterprofiler” package to determine alterations of enriched pathways [19]. Functional enrichment is identified by comparing genes with a predefined group of genes that share localization, pathways, functions, or other features. GO term enrichment analysis was conducted for three sub-ontologies: Cellular component (CC), molecular function (MF), and biological process (BP). *P* ≤ 0.05 was considered statistically significant.

### Protein-protein interaction (PPI) network analysis and hub gene identification

PPI network analysis was conducted using the Search Tool for the Retrieval of Interacting Genes (STRING) online database (STRING, http://string-db.org). An interaction score over 0.4 (medium confidence) was set as threshold value. The PPI network was visualized by employing the “Molecular Complex Detection (MCODE)” plugin in Cytoscape software (version 3.8.2). For detection of typical gene modules, default parameters were set to degree cutoff = 2, node score cutoff = 0.2, k-core = 2, and max. depth = 100. For modules of interest, a score >4 was set as the cutoff threshold. Subsequently, the 10 genes with the highest degree of connectivity were selected. In addition, we selected the 10 highest ranking genes with the MCC algorithm using the “cytohubba” plug-in. These two sets of genes were then intersected to generate a list of hub genes. Expression profiles of hub genes were verified in the GSE112057 cohort using the “limma” package. Modules and hub genes went through GO and KEGG pathway analysis to predict pathological impact.

### Prognostic markers screened with DEGs

To predict prognosis of active SJIA with DEGs, we established a logistic regression model based on data from 77 patients in GSE80060. These patients diagnosed with active SJIA were all treated with canakinumab, a neutralizing monoclonal antibody (mAb) against IL-1beta. Clinical response to treatment was evaluated using the American College of Rheumatology (ACR) response criteria [20] at day 15 after initiation of mAb therapy. In our study, we defined an improvement ≥50% as the cutoff for a “good” versus “poor” response. We allocated all DEGs into least absolute shrinkage and selection operator (LASSO) regression using package “glmnet” [21] to perform a feature selection process to determine the most suitable prognostic markers and compared the expression level of each gene with two different responses. Binary logistic regression model analysis was employed after selecting a set of genes. Subsequently, the receiver operating characteristics (ROC) curve was used to examine the predictive value of selected genes.

### Assessment of immune cells

CIBERSORT (http://cibersort.stanford.edu) is an online tool to distinguish 22 different immune cell types and their respective composition in samples using a deconvolution algorithm. Gene expression data were uploaded to CIBERSORT with LM22 to generate a data matrix. Subsequently, “ggplot2” and “pheatmap” packages were employed to visualize the composition of immune cells. Correlation coefficients were calculated using Pearson correlation analysis and plotted as a heatmap using the “corrplot” package [22]. Immune cells with highest |log2FC| values were selected, correlation with hub genes calculated using Pearson correlation analysis, and results visualized in R software.

### Prediction of potential new drugs

Online database Connectivity Map (CMap; http://www.broadinstitute.org/cmap/) was employed to screen for potential drugs based on the specific gene expression signature. Over- and underexpressed DEGs were uploaded to CMap to obtain a table of predicted agents including enrichment scores. Enrichment scores (ES) ranged from −1 to 1. Drugs with negative enrichment scores have the potential capability to repress pathologically active pathways in SJIA. All drugs with an enrichment score below −0.8 were assessed. *P* ≤ 0.05 was considered statistically significant.

## RESULTS

### Identification of DEGs

**FIGURE 1:**
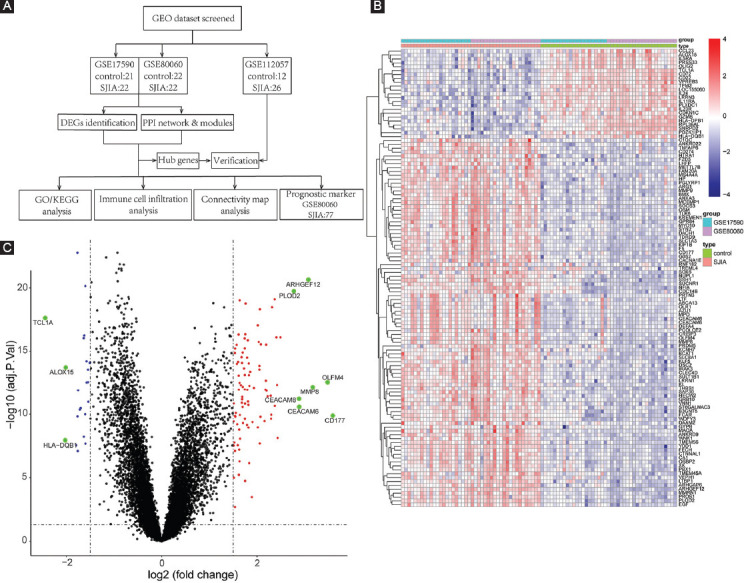
Data retrieving concept and DEG screening. (A) Flow diagram of employed bioinformatic analysis including data retrieving, processing, analyzing, and validation. (B) Heatmap of 118 DEGs for SJIA patients and healthy individuals. (C) Volcano plot of gene expression profile comparing SJIA patients and healthy individuals. Genes with highest |log2FC| values are highlighted in green. GEO: Gene expression omnibus; DEG: Differentially expressed gene; PPI: Protein-protein interaction; SJIA: Systemic juvenile idiopathic arthritis; FC: Fold change.

**FIGURE S1:**
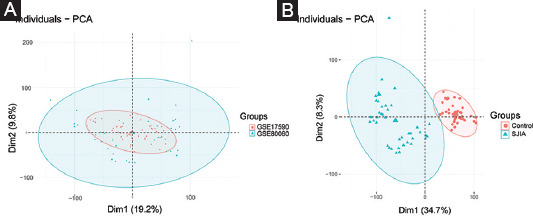
(A) Two-dimensional PCA plots show altered distribution of samples after batch removal of dataset GSE80060 and GSE17590. (B) Group difference between SJIA patients and healthy controls. PCA: Principal component analysis.

The workflows of this study are shown in Figure 1A. We recorded age-matched (median age, SJIA: 9.5, control: 9.0) and gender-matched (female, SJIA: 55%, control: 56%) patients from the two datasets. To gain an overview of altered gene expression of active SJIA, we first identified relevant DEGs. After normalization and removal of batch differences between dataset GSE17590 and GSE80060 (Supplemental Figure 1), we identified a total of 118 DEGs including 94 upregulated and 24 downregulated genes (Figure 1B, Supplemental Table 1). In particular, expression of *CD177*, *OLFM4*, *ARHGEF12*, *MMP8*, *PLOD2*, *CEACAM6*, and *CEACAM8* was highly upregulated (*P* < 0.001, Figure 1C). In contrast, *TCL1A*, *ALOX15*, and *HLA-DQB* were downregulated the most (*P* < 0.001).

**TABLE 1:**
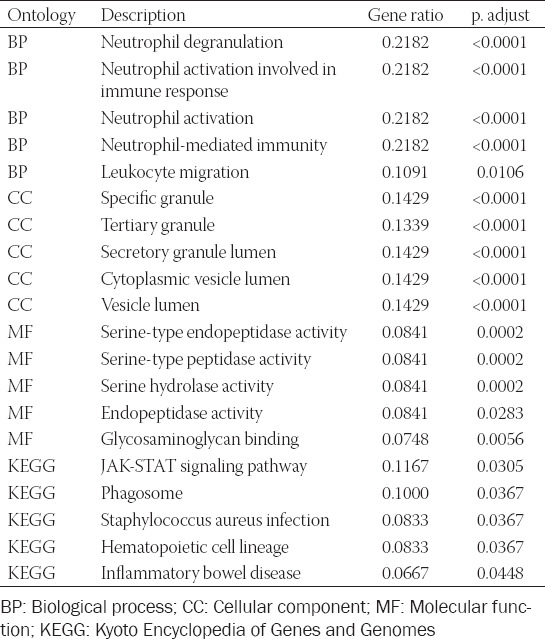
Top 10 enriched terms in each sub-ontology of GO/KEGG analysis of DEGs

### Functional and pathway enrichment analysis

**FIGURE 2:**
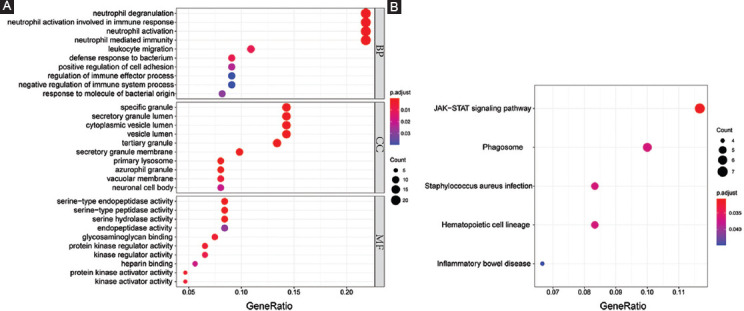
Functional and pathway enrichment analysis. (A) Top 10 results of each sub-term in GO functional enrichment analysis of the DEGs set. (B) Top 10 results of KEGG pathway enrichment analysis of DEGs. GO: Gene ontology; DEG: Differentially expressed gene; KEGG: Kyoto Encyclopedia of Genes and Genomes; BP: Biological process; CC: Cellular component; MF: Molecular function.

Based on the identified DEGs, GO and KEGG analyses were employed to assess the relevant biological functions and pathways. Using GO analysis, the top five terms with highest gene ratio in each sub-ontology were identified (Table 1 and Figure 2A). For biological processes, “neutrophil degranulation,” “neutrophil activation involved in immune response,” “neutrophil activation,” “neutrophil-mediated immunity,” and “leukocyte migration” were ranked highest. For cellular components, gene ratio was highest in “specific granule,” “secretory granule lumen,” “cytoplasmic vesicle lumen,” “vesicle lumen,” and “tertiary granule.” For molecular function, “serine-type endopeptidase activity,” “serine-type peptidase activity,” “serine hydrolase activity,” “glycosaminoglycan binding,” and “protein kinase regulator activity.” Five statistically significant KEGG pathways were identified, among which the Janus kinase/signal transduction and activator of transcription (JAK-STAT) signaling pathway had the highest gene ratio (Table 1 and Figure 2B).

### Identification of PPI modules and hub genes

**FIGURE 3:**
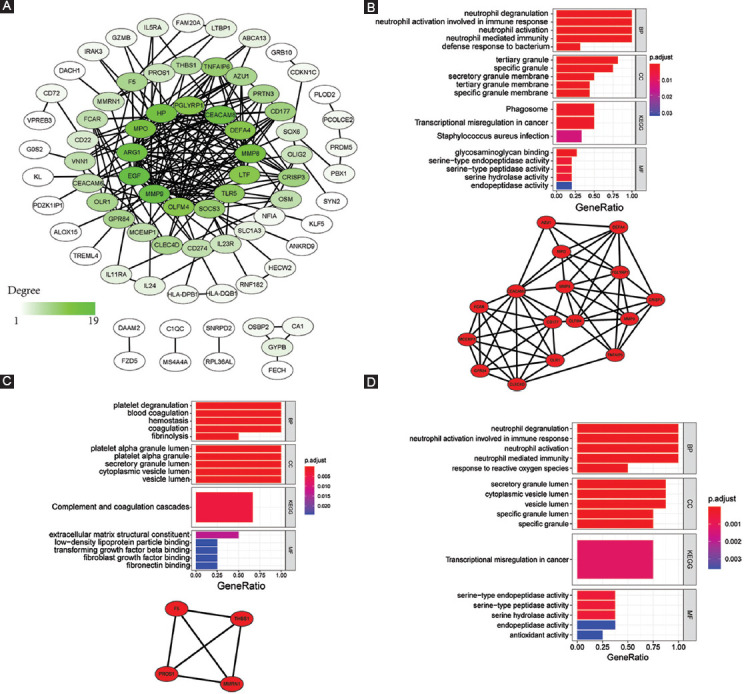
PPI network. (A) Overview of PPI network of DEGs in SJIA patients and healthy controls. Color darkness intensity represents gene connectivity. (B) PPI network and GO/KEGG analysis of gene set of module A. (C) PPI network and GO/KEGG analysis of gene set of module B. (D) GO/KEGG analysis of the hub gene set. BP: Biological process; CC: Cellular component; MF: Molecular function; KEGG: Kyoto Encyclopedia of Genes and Genomes; PPI: Protein-protein interaction; DEG: Differentially expressed gene; SJIA: Systemic juvenile idiopathic arthritis; GO: Gene ontology.

**FIGURE S2:**
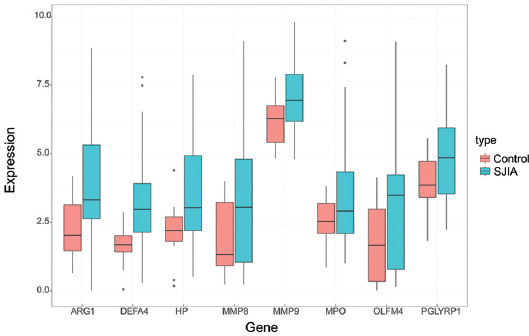
Validation of expression level of hub genes in GSE112057 dataset.

PPI analysis was used to decode the underlying interactions between proteins and the molecular pathogenesis. We obtained a PPI network with a total of 117 nodes with 199 edges from the STRING database and Cytoscape software (Figure 3A). While each node represents one gene, the edges represent an interaction between two genes. In total, five modules were identified using MCODE (Table 2). The two modules with an MCODE score ≥4 (module A and B) were analyzed further (Figure 3B-C). The genes associated with these two modules were all upregulated compared to the healthy controls. After intersecting the gene sets generated using the plug-in “cytohubba,” we found a hub gene set of eight upregulated genes including *ARG1*, *DEFA4*, *HP*, *MMP8*, *MMP9*, *MPO*, *OLFM4*, and *PGLYRP1* (Table 2). No downregulated genes were found. The GSE112057 dataset was used to confirm these findings (Supplemental Figure 2). Equally, the same eight genes were significantly upregulated in SJIA patients (Table 3).

**TABLE 2:**
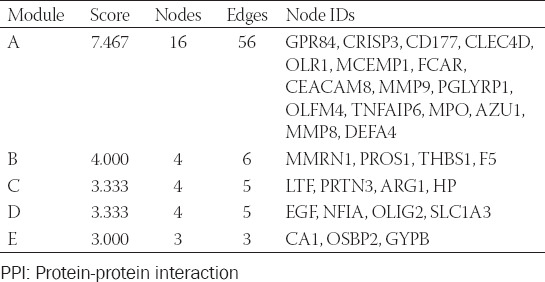
Properties and node IDs of modules identified by PPI network analysis

**TABLE 3:**
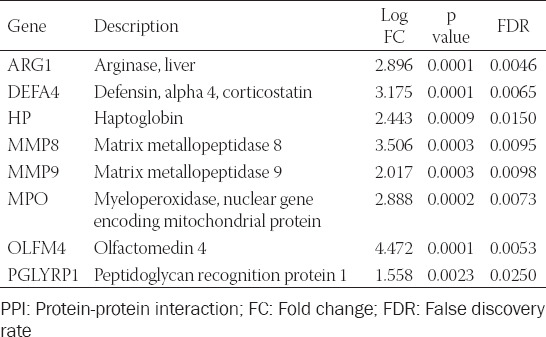
Hub genes identified by PPI network analysis

Functional enrichment analysis demonstrated these eight hub genes to be mainly involved in neutrophil function, serine peptidase pathways, and misregulation in cancer (Figure 3D). Module A was particularly related to neutrophil function with a gene ratio of 1.0 (Figure 3B). Of note, the whole DEG profile, module A, and hub gene set were consistently related to neutrophil function. In contrast, module B was associated with hemostatic and secretory functions (Figure 3C).

### Screening and testing markers with prognostic value

**FIGURE 4:**
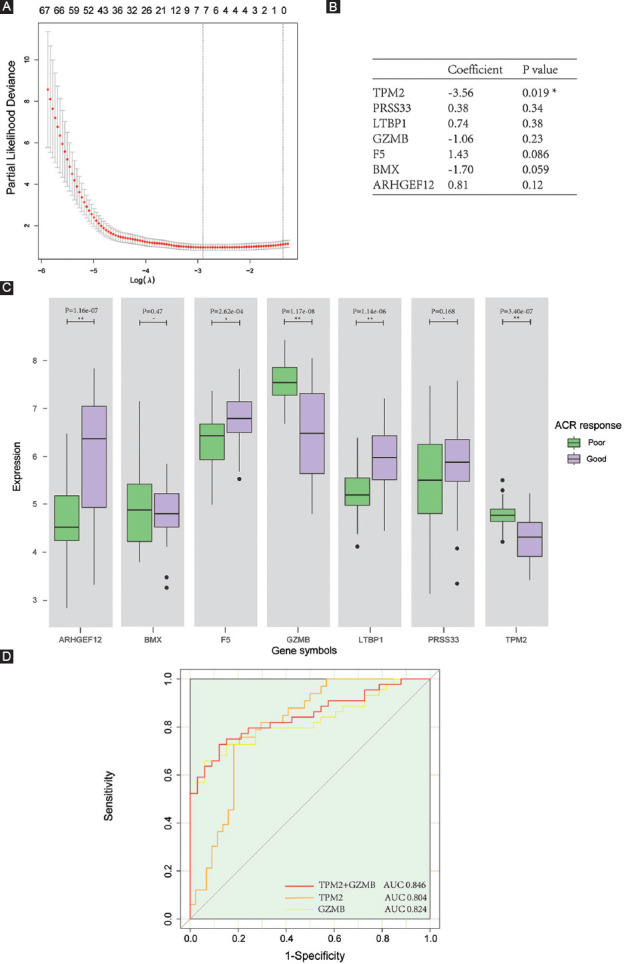
Prognostic markers screening and testing. (A) Partial likelihood deviance plot for LASSO regression analysis. (B) Binary logistic regression model with most potential genes. (C) Different expression patterns compared between two response types. (D) ROC curve for testing prognostic value. ACR: American College of Rheumatology.

Using ACR criteria, 44 patients with “good” and 33 patients with “poor” response to treatment were identified. We input all 118 DEGs into the LASSO regression algorithm and used the minimum value of lambda (lambda.min) as the cutoff (Figure 4A). A total of seven genes were screened out as potential prognostic markers, including *TPM2*, *PRSS33*, *LTBP1*, *GZMB*, *F5*, *BMX*, and *ARHGEF12*. Using a binary logistic regression model, significant correlation to treatment response was only found for *TPM2* (*P* = 0.019; coefficient = −3.56; Figure 4B). Five of the analyzed genes showed differential expression levels for “good” and “poor” clinical outcome (Figure 4C). ROC analysis for these genes suggested a good predictive value of *TPM2* for short-term prognosis with an area under the curve (AUC) of 0.803 and *GZMB* with an AUC of 0.824. A combination of *GZMB* and *TPM2* improved AUC to 0.846 (Figure 4D).

### Proportion of immune cells and correlation

**FIGURE 5:**
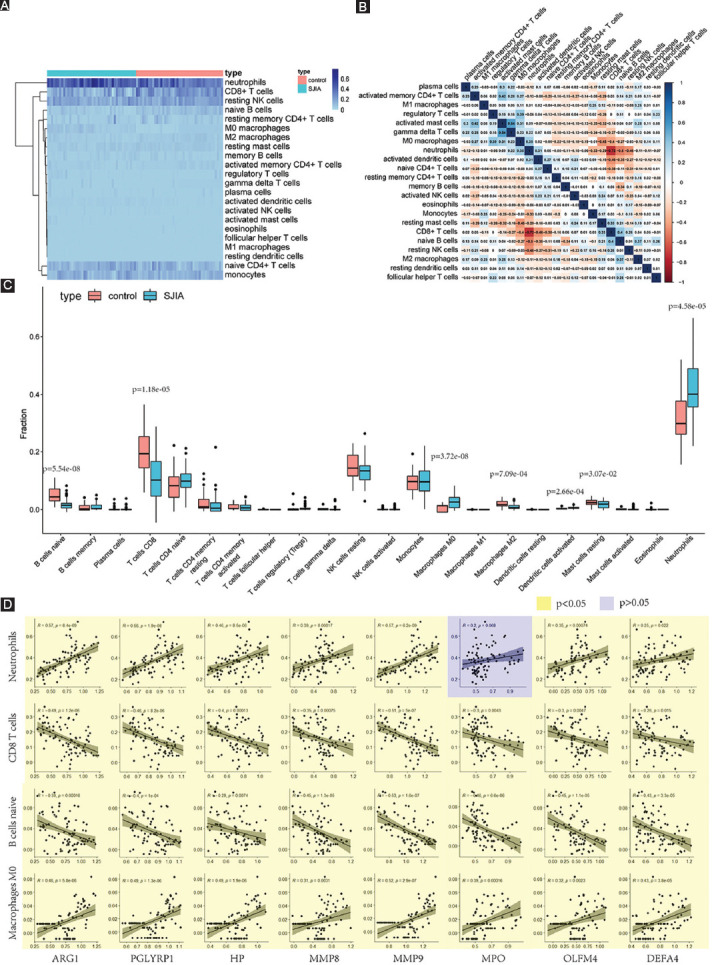
Comparison of 22 different immune system cells in peripheral blood specimens. (A) Heatmap of immune system cells in SJIA patients and healthy controls. (B) Correlation heatmap plot of 22 immune system cells. Color darkness intensity represents correlation between two cells. (C) Boxplot distribution of immune system cells. Significant differences were marked with p values. (D) Linear regression plot between eight hub genes and the four immune system cells with largest shift in prevalence in SJIA. SJIA: Systemic juvenile idiopathic arthritis.

Immune reactivity plays an essential role in SJIA. Therefore, we investigated the constitution of the immune landscape surrounding the disease development. Relative numbers of 22 types of immune cells were calculated using the CIBERSORT algorithm (Figure 5A). Compared to the composition in healthy individuals, neutrophils, M0 macrophages, and activated dendritic cells were upregulated, while the percentage of resting mast cells, M2 macrophages, naïve B cells, and CD8+ T cells was decreased (Figure 5C). Of these cells, neutrophils were most prominently upregulated (*P* < 0.001) and CD8+ T cells (*P* < 0.001) most severely downregulated. Correlation coefficients for all 22 types of immune cells were calculated and displayed as a heat map (Figure 5B). Activated mast cells and gamma delta T cells showed the strongest positive correlation (r = 0.54, *p* < 0.001), while neutrophils and CD8+ T cells were most negatively correlated (r = –0.72, *p* < 0.001). Correlation analysis also demonstrated significantly positive correlation for the proportion of neutrophils and M0 macrophages with expression of all hub genes except *MPO*. In addition, the percentage of CD8+ T cells and naïve B cells was significantly negatively correlated with expression of all hub genes (Figure 5D).

### Prediction of potentially effective drugs against active SJIA

A list of potential small molecular drugs targeting the up- and downregulated DEGs was generated. In total, eight drugs were identified: Sulindac sulfide (ES = −0.975, *p* < 0.001), phenyl biguanide (ES = −0.963, *p* < 0.001), (-)-catechin (ES = −0.957, *p* < 0.001), splitomicin (ES = −0.903, *p* < 0.001), methocarbamol (ES = −0.901, *p* = 0.002), Gly-His-Lys (ES = −0.861, *p* = 0.005), phenanthridinone (ES = −0.847, *p* < 0.001), and hycanthone (ES = −0.81, *p* = 0.003) (Table 4).

**TABLE 4:**
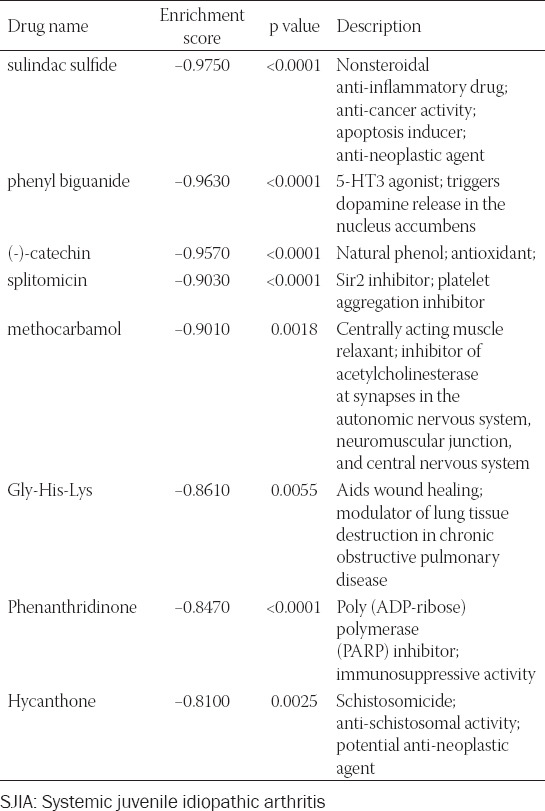
Characteristics of the most significant predicted drugs for potential treatment of SJIA

## DISCUSSION

Despite treatment, SJIA is a refractory and recurrent disease that is associated with systemic inflammation and high mortality rates [8,15,23]. The identification of molecular changes and alterations in immune profile composition is paramount for a better understanding of the pathomechanisms involved and may provide new therapeutic targets [10,24].

GO enrichment demonstrated DEGs enrichment in neutrophil-related processes, including neutrophil degranulation, neutrophil-mediated immunity, and neutrophil activation. In accordance with our results, previous research found neutrophilia to be closely linked to the activity of SJIA [6,11,25]. Neutrophils are major innate immune effector cells and play a key role in the response against microorganisms [26]. Immunological functions including degranulation and excretion of reactive oxygen species are upregulated in these effector cells in inflammation [26]. Reverting these sepsis-like features of neutrophils by current treatment strategies showed satisfactory outcomes in patients with SJIA [4]. In active SJIA, neutrophil function is significantly upregulated and outlasts clinical symptoms suggesting neutrophils to play a decisive role in long-term disease progression [6]. In addition, in contrast to other JIAs, the prevalence of suppressive CD16+/CD62L(dim) neutrophils was lower in patients with clinically inert SJIA suggesting neutrophil heterogeneity and unique molecular pathomechanisms to be involved [6]. Considering the significant role of the immune system in the pathogenesis of SJIA, we explored the relative prevalence of 22 subtypes of immune cells. Confirming previous results, we found elevated prevalence of neutrophils [6,25]. In contrast to a previous study, we found lower levels of CD8+ T cells [27]. We hypothesize that this difference is due to a dynamic change in cell prevalence depending on disease progression. Silvestre-Roig et al. suggested CD16+/CD62L(dim) neutrophil-driven suppression of T-cell proliferation through integrins and ROS production as a potential mechanism [28]. Further evidence is lent to this theory by our results showing negative correlation of neutrophil and CD8+ T-cell prevalence suggesting disease stage-dependent regulatory interplays between innate and adaptive immune cells.

Previous research demonstrated neutrophil-related protease activity to be regulated by the JAK-STAT pathway [29]. Using KEGG analysis, the JAK-STAT pathway was identified as the most enriched pathway. Similarly, previous research using SJIA models and ingenuity pathway analysis suggested inhibition of the JAK-STAT pathway to be a viable therapy and an ongoing randomized controlled trial (NCT03000439) is anticipated to provide therapeutical evidence for patients with SJIA [25,30,31].

In total, we identified eight upregulated hub genes in patients with active SJIA: *HP*, *MPO*, *MMP8*, *MMP9*, *ARG1*, *OLFM4*, *DEFA4*, and *PGLYRP1*. We postulate these genes to be significantly involved in the inflammatory environment and subsequent disease progression in patients with active SJIA. HP encodes haptoglobin, which binds free hemoglobin in the plasma by forming a haptoglobin-hemoglobin complex that can be taken up by CD163+ macrophages [32]. In accordance with our results, *HP* was found to be overexpressed in peripheral blood samples in SJIA patients with subclinical MAS [33]. Together with the MMP family, MPO mediates antimicrobial activity and pro-inflammatory response by induction of oxidative tissue damage and neutrophil respiratory burst [34,35]. Despite its localized effectiveness, we found expression of *MPO*, *MMP8*, and *MMP9* to be elevated in systemically affected SJIA patients. In accordance with these results, the previous studies have suggested elevated expression of *MPO*, *MMP8*, and *MMP9* in several inflammatory morbidities such as SJIA [25,31,36]. Further, *MMP9* has been suggested as a prognostic plasma biomarker in patients with SJIA [37]. *ARG1*, arginase-encoding gene, was reported to be upregulated in the peripheral blood and cancer tissue of various cancer patients [38-40]. In tumor-associated myeloid cells, upregulation of *ARG1* has been shown to reduce inflammation by suppressing T-cell proliferation through arginine deprivation [41,42]. Moreover, *ARG1* has been shown to be upregulated in autoimmune disease such as rheumatoid arthritis [43]. In SJIA, *ARG1* was reported to be linked to anti-inflammatory M2 macrophage polarization [44] – however, there is lack of more detailed knowledge on its role in disease progression. We also found increased expression of *OLFM4*, *DEFA4*, and *PGLYRP1*, which encode olfactomedin-4, defensin alpha 4, and peptidoglycan recognition protein 1, respectively. Albeit these genes have been linked to autoimmune diseases [45-47], elevated expression in SJIA has not been reported and their function is yet to be determined. Upregulation of both pro- and anti-inflammatory genes indicates neutrophils to play a complex, dual role in SJIA.

GO and KEGG enrichment analysis was repeated for modules and the hub gene set to identify their own specific effects and to exclude potentially confounding genes. Conversely, KEGG analysis on module B related to hemostatic and secretory functions. Correlation analysis demonstrated all eight upregulated genes described above to positively correlate with neutrophil and M0 macrophage and to negatively correlate with CD8+ T cells and naïve B cells. In consideration of these results and our hub genes analysis, alteration of neutrophil prevalence and function appears to be a decisive pathological deviation in SJIA. Further, analysis of neutrophil heterogeneity and function in SJIA is necessary to improve our understanding of SJIA pathogenesis and to potentially develop novel neutrophil-specific treatment approaches.

To bring our results into a translational context, we investigated markers to predict the prognosis and treatment response. Canakinumab is often prescribed at an early stage of SJIA yet is not universally effective in all patients [48]. In our research, we utilized LASSO regression and a binary logistic regression model to identify predictive markers for treatment response to canakinumab. We found *TPM2* to have a significant predictive value. Tropomyosin 2, encoded by *TPM2*, is an intracellular protein isoform in the tropomyosin family that binds to and stabilizes actin filaments in muscle fibers. However, its role in SJIA is unknown. In addition, we found *GZMB* encoding granzyme B, a serine proteinase secreted by cytotoxic T cells and natural killer cells, to be a promising predictive marker. *GZMB* has been linked to progression of JIA previously, even though it was not screened out by logistic regression model [49]. Both markers were significantly decreased in patients with a good treatment response and can potentially be utilized clinically.

Using CMap, we evaluated potential drugs with activity against the differentially expressed genes found in this study. Of the identified drugs, sulindac sulfide, (-)-catechin, and phenanthridinone were the most promising based on their known anti-inflammatory properties. Phenyl biguanide, splitomicin, methocarbamol, Gly-His-Lys, and hycanthone have not been reported to be effective against inflammation. However, further research on these drugs might provide novel therapeutic approaches. Sulindac sulfide is a non-selective anti-inflammatory drug that inhibits the chlorinating activity of MPO [50]. In addition, it displays mild COX-2 inhibiting effects and possesses potent ability to decrease ROS levels [51,52]. Catechin has been shown to inhibit production of pro-inflammatory factors IL-1, TNF-alpha, and prostaglandin E2 in adjuvant arthritis [53]. However, there is a paucity of knowledge on the specific effect of the (-)-catechin enantiomer. Phenanthridinone is an inhibitor of the nuclear enzyme poly(ADP-ribose) polymerase-1 (PARP-1) [54]. In a rodent acute lung inflammation model, phenanthridinone downregulated MPO activity and subsequent inflammatory cytokine excretion [55]. In addition, phenanthridinone was shown to suppress neutrophil infiltration in local inflammation [54].

## CONCLUSION

This study provides a new insight into molecular and cellular pathogenesis of active SJIA and highlights potential targets for further research. We used bioinformatic analyses to demonstrate the essential role of neutrophils and to identify hub genes involved in the pathogenesis of active SJIA. In addition, we identified *TPM2* and *GZMB* as potential prognostic markers.

The observations in this study also have implications for future investigations. For a deeper understanding of disease progression and to identify new molecular targets, the involved and highly complex cytokine-mediated cell-cell interactions will have to be unraveled in more detail. In our study, we identified several new molecular agents as potential therapeutic candidates for patients with active SJIA. Whether these drugs can successfully translate into clinical treatment regimens remain to be investigated.

## Figures and Tables

**TABLE S1:**
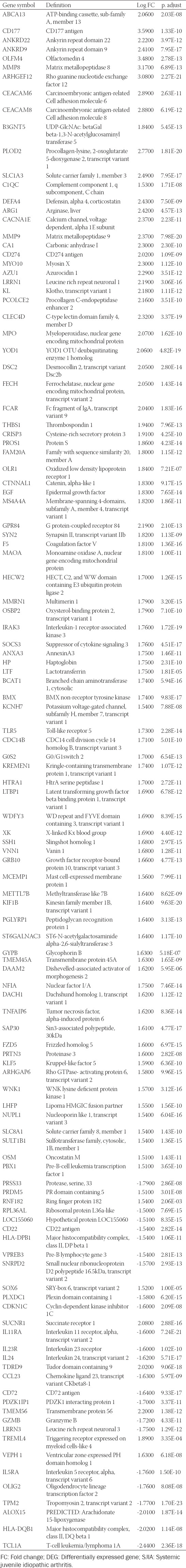
Full list of the 118 DEGs, including 94 upregulated and 24 downregulated genes
